# Circadian-Based Optimization of Pharmacotherapy in Chronic Diseases: Integrating Pharmacokinetics, Pharmacodynamics, and Disease Rhythm

**DOI:** 10.7759/cureus.116920

**Published:** 2026-09-25

**Authors:** Monashri C, Siddhant Mehrotra, Nidhi Srivastava, Ayush Jain, Rakesh Dixit

**Affiliations:** 1 Pharmacology and Therapeutics, King George's Medical University, Lucknow, IND

**Keywords:** chronopharmacokinetics, chronopharmacology, chronotherapy, circadian rhythm, medication timing, precision chronomedicine

## Abstract

Circadian rhythms are endogenous, approximately 24-hour biological cycles that regulate physiological processes, including cardiovascular function, metabolism, immune activity, endocrine signaling, and sleep-wake patterns. These rhythms significantly influence disease pathophysiology and therapeutic responses, making biological time an important determinant of treatment outcomes. Several chronic diseases, including cardiovascular disorders, asthma, rheumatoid arthritis, metabolic syndrome, and psychiatric conditions, exhibit predictable circadian variations in symptom severity and disease activity. Simultaneously, circadian oscillations affect pharmacokinetic processes such as drug absorption, distribution, metabolism, and excretion, as well as pharmacodynamic mechanisms involving receptor sensitivity and cellular responsiveness. As a result, identical drug doses administered at different times may produce considerable differences in efficacy and toxicity.

This review analyzes the role of circadian biology in optimizing pharmacotherapy through the integration of chronopharmacology and chronotherapy. It discusses the molecular basis of circadian regulation and its influence on physiological systems, pharmacokinetics, and pharmacodynamics. Disease-specific chronotherapeutic applications in cardiovascular, metabolic, endocrine, respiratory, rheumatologic, psychiatric, and oncologic disorders are highlighted. Clinical examples, including evening statin administration, timed bronchodilator use, and modified-release glucocorticoid therapy, highlight the practical benefits of circadian-based therapies. However, evidence remains variable across therapeutic areas, with some chronotherapeutic strategies, such as bedtime antihypertensive dosing, showing conflicting results in recent clinical trials. Further research is needed to define optimal timing recommendations and facilitate wider clinical adoption. Throughout, the strength of evidence is appraised to distinguish chronotherapeutic strategies with sufficient support for routine clinical use from those that remain experimental. The review also discusses emerging developments in chronopharmacokinetic modeling, advanced drug delivery systems, wearable monitoring devices, and precision chronomedicine. Despite promising evidence, broader implementation remains limited by variability in circadian patterns, lack of awareness, and insufficient standardized guidelines. Integrating circadian principles into pharmacotherapy and, ultimately, combining precision chronotherapy with lifestyle-based strategies may offer a promising direction for improving therapeutic precision, disease prevention, and patient outcomes.

## Introduction and background

Circadian rhythms are inbuilt, approximately 24-hour cycles regulated by the central clock in the hypothalamic suprachiasmatic nucleus (SCN) and peripheral biological clocks that synchronize physiological processes with environmental stimuli such as the light-dark cycle [1]. These rhythms are controlled by internal biological clocks involving transcriptional-translational feedback loops that regulate gene expression across multiple organ systems [2]. Circadian rhythms play a vital role in regulating cardiovascular activity, metabolism, immune responses, and neuroendocrine signaling [3].

Inflammation and immune activity exhibit significant circadian variation, influencing the pathophysiology of many chronic diseases [4]. For example, respiratory disorders such as asthma worsen at night [5], rheumatoid arthritis shows morning stiffness and pain [6], and cardiovascular events like myocardial infarction and stroke occur more frequently in the early morning [7]. Circadian rhythms also regulate glucose metabolism and insulin sensitivity, contributing to metabolic disorders [8]. Disruption of these rhythms in psychiatric disorders alters sleep, mood, and treatment response [9].

Circadian rhythms play an important role in chronopharmacokinetics by influencing drug absorption, distribution, metabolism, and elimination (ADME) through time-dependent physiological changes [10,11]. For example, hepatic cytochrome P450 (CYP) enzyme activity and intestinal drug transporter (P-glycoprotein) expression exhibit circadian variations, leading to variability in drug plasma concentrations [2]. Pharmacodynamic responses are also subject to circadian modulation due to variations in receptor expression, target tissue sensitivity, and downstream signaling pathways [3,12]. Hence, identical drug doses administered at different times of the day may produce different therapeutic and adverse effects [13].

This understanding has led to the development of chronotherapy, an approach that aligns drug administration with biological rhythms to enhance efficacy and minimize toxicity [14]. Clinical applications include bedtime statin administration in cardiovascular disease (CVD), time-modified glucocorticoid therapy in rheumatoid arthritis, and optimized bronchodilator use in asthma [15,16]. However, despite strong mechanistic rationale and encouraging clinical evidence in selected settings, chronotherapy remains underutilized in routine clinical practice [17].

Therefore, this review aims to integrate current evidence on circadian regulation of pharmacokinetics and pharmacodynamics and evaluate its implications for optimizing pharmacotherapy across major chronic diseases. Despite a substantial supporting literature, the clinical adoption of chronotherapy remains limited: the timing of administration is seldom considered in routine prescribing, and timing recommendations are inconsistently reflected in clinical guidelines. An updated synthesis is timely because the evidence base has shifted appreciably in recent years, large randomized trials have reshaped the case for bedtime antihypertensive dosing, immuno-oncology has emerged as a new area of circadian interest, and advances in drug-delivery and circadian-biomarker technologies have broadened what is feasible. The distinct contribution of this review is therefore to integrate these mechanistic, clinical, and technological developments while explicitly grading the strength of evidence for each application, distinguishing strategies ready for clinical consideration from those that remain experimental, and to translate this appraisal into practical guidance for clinicians.

## Review

Methodology

This article is a narrative review, and the supporting literature was identified through a structured, non-systematic search of PubMed and Google Scholar for peer-reviewed, English-language publications available up to April 2026. Search terms combined the core concepts "circadian rhythm", "chronotherapy", "chronopharmacology", "chronopharmacokinetics", "medication timing", and "precision chronomedicine" with disease-specific terms such as hypertension, asthma, rheumatoid arthritis, diabetes mellitus, and cancer. Priority was given to original research, randomized controlled trials, systematic reviews, and authoritative reviews, and the reference lists of selected articles were screened manually to identify additional relevant sources. Studies were included on the basis of their relevance to the circadian regulation of pharmacokinetics, pharmacodynamics, and disease-specific chronotherapeutics, with preference given to recent and informative work. As this is a narrative rather than a systematic review, the evidence was synthesized qualitatively and organized by physiological system and disease area; a formal quantitative meta-analysis and structured risk-of-bias assessment were not undertaken. Where relevant, the relative strength of the underlying evidence ranging from randomized controlled trials to observational or mechanistic data was nonetheless considered in interpreting each application. Where studies reported conflicting findings, most notably on the timing of antihypertensive therapy, greater weight was given to larger, methodologically rigorous randomized trials than to smaller or open-label studies, and the reasons for discordant results were examined in the relevant sections.

Circadian biology as a determinant of pharmacotherapy

Temporal Determinants of Drug Response and Therapeutic Outcomes

Modern pharmacotherapy has achieved remarkable precision in selecting appropriate drugs and determining optimal doses; however, the timing of drug administration remains an often-underappreciated variable. This overlooks the fundamental principle that human physiology operates within a circadian framework [1,3]. These observations form the basis of chronopharmacology, the study of how circadian rhythms influence drug pharmacokinetics and pharmacodynamics, and chronotherapy, which applies this knowledge by aligning drug administration with biological rhythms to maximize efficacy and minimize toxicity [3,8].

Molecular Basis of Circadian Rhythms and Systemic Temporal Regulation

Circadian regulation involves a network of central and peripheral molecular clocks that coordinate near 24-hour biological rhythms [1,3]. The central clock synchronizes these peripheral oscillators through neural (autonomic nervous system) and hormonal signals (e.g., glucocorticoid and melatonin), maintaining synchronization across organ systems. At the molecular level, circadian rhythmicity is regulated by core clock genes such as CLOCK, BMAL1, PER, and CRY, which generate feedback inhibition cycles that drive rhythmic expression of genes involved in metabolic enzymes, ion channels, hormone receptors, and cytokine production pathways [3]. As a result, thousands of genes exhibit circadian oscillations [2].

Circadian regulation of physiological systems

Cardiovascular System

Blood pressure, heart rate, vascular tone, and platelet activity follow predictable daily rhythms. A notable feature is the morning surge in sympathetic activity and catecholamine release, accompanied by increased blood pressure and prothrombotic tendency driven by peak levels of plasminogen activator inhibitor-1 (PAI-1) and reduced fibrinolytic activity [18]. This explains why adverse cardiovascular events such as myocardial infarction, stroke, and sudden cardiac death occur more frequently in the early morning hours. Therefore, the transition from a nocturnal rest mode characterized by parasympathetic dominance and physiological blood pressure dipping to heightened physiological activity in the morning creates a window of vulnerability for acute ischemic events [7].

Metabolic System

Glucose tolerance, insulin sensitivity, and hepatic glucose production vary across the day. Insulin sensitivity is higher during the morning and declines in the evening, while hepatic gluconeogenesis (driven by enzymes like PEPCK) and cholesterol synthesis increase overnight [8,19,20]. Similarly, pancreatic β-cell function and incretin (e.g., glucagon-like peptide-1 (GLP-1)) secretion exhibit distinct diurnal rhythmicity regulated by the intestinal environment and the core clock gene BMAL1. Endogenous GLP-1 levels peak around 2:00 PM, while insulin secretion and β-cell responsiveness are highest between noon and 6:00 PM and decline to their lowest levels between midnight and 6:00 AM [19,20]. Disruption of these rhythms, such as in shift workers or individuals with irregular sleep patterns, leads to metabolic dysregulation, insulin resistance, and an increased risk of diabetes and obesity [20].

Immune and Inflammatory Systems

Immune cell trafficking (such as leukocyte recruitment from the bone marrow), cytokine release, and inflammatory responses all exhibit rhythmic variation controlled by clock genes like BMAL1 and REV-ERBα within macrophages and T cells [5]. Pro-inflammatory cytokines, such as tumor necrosis factor-alpha (TNF-α), IL-1β, and IL-6, often peak during the night and early morning, coinciding with symptom exacerbation in many inflammatory diseases [4,21]. Importantly, this relationship is bidirectional. While circadian rhythms regulate immune function, inflammatory signals (such as elevated TNF-α) can also disrupt local circadian clocks by interfering with clock-gene transcription, creating a self-amplifying loop of circadian disruption and disease progression [4].

Respiratory System

In respiratory diseases such as asthma, circadian fluctuations in airway caliber, bronchial reactivity, and inflammatory mediator levels contribute to nocturnal airway narrowing, increased inflammation, and early morning exacerbations [5,22]. These changes are influenced by rhythmic neutrophil recruitment and a nocturnal shift toward parasympathetic autonomic tone [22].

Neuropsychiatric Regulation

Circadian rhythms play a central role in regulating sleep-wake cycles, mood, and cognitive function via the rhythmic release of neurotransmitters and neuroendocrine hormones, including melatonin, serotonin, dopamine, and cortisol. Disruption of circadian timing is associated with psychiatric disorders, including depression, bipolar disorder, and anxiety [9].

Circadian control of pharmacokinetics (PK)

Pharmacokinetics describes what the body does to a drug, including its absorption, distribution, metabolism, and excretion (ADME). Each of these processes is influenced by circadian rhythms.

Gastrointestinal physiology shows circadian variation in gastric pH, motility, and blood flow, influencing drug absorption and oral bioavailability. Higher nocturnal acid secretion and slower evening gastric emptying may delay peak plasma concentrations of drugs administered later in the day [2,10]. Lipophilic drugs are generally absorbed faster in the morning than in the evening due to a combination of faster gastric emptying and higher gastrointestinal blood perfusion early in the day [10]. The absorption of drugs is also influenced by intestinal transporters (like Slc15a1/PepT1 and Slc22a4/Octn1) and efflux pumps (like Abcb1), which exhibit 24-hour rhythmic expression and directly cause temporal variations in systemic drug exposure [2,11].

Drug distribution depends on plasma protein binding and tissue perfusion, both of which show circadian variation [1]. Driven by the sympathetic and parasympathetic systems, overall blood flow and local tissue perfusion increase during the daytime and decrease at night. The concentrations of human plasma proteins (such as albumin and α1-acid glycoprotein) increase during the day. As a result, the unbound fraction of drugs increases during nocturnal rest [10]. For instance, the unbound fraction of diazepam is lower following a morning administration [11].

Drug-metabolizing enzymes, particularly CYP450 isoenzymes, exhibit circadian variation [2,11]. Phase I oxidative metabolism is generally higher during the active phase (daytime), whereas phase II sulfate conjugation is more prominent during the rest phase (nighttime), resulting in time-dependent changes in drug clearance, half-life, and active metabolite formation. Drug elimination varies depending on the time of administration [10]. Glomerular filtration rate (GFR) and overall renal blood flow peak during the daytime and drop to their lowest levels at night [2,11]. Renal clearance by glomerular filtration is generally higher during the day, while urinary pH becomes more acidic at night and more alkaline during the day [3,8]. Nocturnal urinary acidification increases the non-ionized fraction of acidic drugs, enhancing their passive renal reabsorption at night [8,11]. Biliary excretion also shows variation due to rhythmic bile flow and expression of efflux pumps such as P-gp and MRP2. Thus, biliary excretion of some drugs peaks during the morning or early rest phase [10,11,23].

Circadian regulation of pharmacodynamics (PD)

If pharmacokinetics determines how much drug reaches the target, pharmacodynamics determines the biological response elicited at that site. Circadian rhythms influence pharmacodynamics through variation in receptor expression, changes in signal transduction pathways, and alterations in cellular responsiveness. For example, the sensitivity of target tissues to drugs may vary across the day, leading to differences in efficacy and toxicity even when plasma drug levels remain constant [3,12].

Integrating PK-PD with circadian biology

The true complexity of chronopharmacology emerges when PK and PD are considered together. Circadian rhythms simultaneously influence drug concentration (PK) and drug effect (PD). A drug may achieve higher plasma concentrations at one time due to favorable pharmacokinetic conditions, while at another time, enhanced receptor sensitivity may amplify its pharmacodynamic effect. The optimal dosing time is therefore not random but represents a point of alignment between drug kinetics, target sensitivity, and disease activity [13]. For instance, peak drug exposure should ideally coincide with the specific phase of the 24-hour cycle when target pathophysiological pathways are most active, thereby maximizing chronoefficacy and minimizing chronotoxicity [2].

By recognizing biological time as a determinant of drug response, pharmacotherapy evolves into a 4D pharmacology model, where drug, dose, patient, and time collectively determine therapeutic outcomes [3,12].

Chronotherapy: from concept to clinical practice

Having established the biological and pharmacological foundations of circadian regulation, the following sections examine how these principles translate into disease-specific therapy, aligning peak drug activity with periods of highest physiological risk [2,12].

The clinical applications discussed below vary considerably in the strength of supporting evidence. While some, such as evening dosing of short-acting statins, modified-release prednisone for rheumatoid arthritis, and selected chronomodulated chemotherapy regimens, are supported by randomized trials or regulatory approval, many others, including bedtime antihypertensive dosing and most metabolic, psychiatric, and immuno-oncologic timing strategies, remain investigational, relying primarily on mechanistic rationale, observational data, or small preliminary studies. This distinction is maintained throughout the review to reflect their current level of clinical readiness.

Chronotherapy in cardiovascular diseases

Antihypertensives and Circadian Blood Pressure Regulation

Night-time administration of angiotensin-converting enzyme (ACE) inhibitors and angiotensin receptor blockers (ARBs) has been proposed to improve nocturnal blood pressure control and restore physiological dipping patterns by targeting nocturnal renin-angiotensin-aldosterone system (RAAS) activity [19,20]. Early studies, including the MAPEC and Hygia trials, reported substantial reductions in major adverse cardiovascular events with bedtime antihypertensive dosing [20,24,25]. However, subsequent large randomized trials such as TIME and BedMed found no significant differences in cardiovascular outcomes, mortality, or safety between morning and evening administration [26,27]. In TIME (approximately 21,000 patients), the primary cardiovascular endpoint occurred in 3.4% of the evening group versus 3.7% of the morning group (hazard ratio: 0.95, 95% CI: 0.83-1.10), and BedMed (3,357 patients) likewise found no difference (adjusted hazard ratio: 0.96, 95% CI: 0.77-1.19). These conflicting results likely reflect differences in trial quality. MAPEC and Hygia were open-label trials from a single research group, and their unusually large reported benefits [24,25], together with concerns about the conduct and data integrity of Hygia [28], have cast doubt on their reliability. The larger, blinded-endpoint TIME and BedMed trials are therefore regarded as more robust, and their neutral findings carry greater weight. Current evidence therefore favors individualized dosing schedules based on patient characteristics, blood pressure patterns, and treatment adherence rather than routine bedtime dosing [14,29].

Antiplatelet Chronotherapy

To counteract the morning peak in platelet aggregability and reduced fibrinolytic activity, the chronotherapy of low-dose aspirin has attracted clinical interest [18]. Because aspirin has a short pharmacokinetic half-life, evening administration ensures that maximum COX-1 inhibition coincides with the early morning surge in platelet activation [1]. Some clinical studies suggest that bedtime aspirin reduces morning platelet reactivity more effectively than upon-awakening administration, although these findings are based largely on surrogate markers rather than clinical endpoints [4,18].

Circadian Regulation of Lipid Metabolism and Statin Therapy

Cholesterol synthesis in the liver peaks during the night, driven by circadian regulation of HMG-CoA (3-hydroxy-3-methyl-glutaryl-coenzyme A) reductase. This explains why short-acting statins, such as simvastatin, are more effective when administered in the evening [4,30]. Administering statins during the period of maximal endogenous cholesterol synthesis enhances therapeutic efficacy without increasing drug dosage [19]. However, due to pharmacokinetic differences, long-acting statins (e.g., atorvastatin and rosuvastatin) maintain sustained plasma concentrations across 24 hours, and thus their efficacy is less dependent on dosing time [8].

Personalized Cardiovascular Chronotherapy

Recent advances in circadian medicine indicate that optimal drug timing may vary among individuals due to differences in chronotype, sleep-wake patterns, lifestyle, and comorbidities (such as chronic kidney disease or diabetes), influencing circadian physiology and therapeutic response [29]. For instance, relying on 48-hour ambulatory blood pressure monitoring (ABPM) allows clinicians to accurately identify a patient's specific blood pressure phenotype (e.g., non-dipper or extreme dipper) rather than relying on isolated daytime clinic measurements [7,14]. These insights have led to the concept of personalized chronotherapy, where treatment schedules are tailored to an individual’s biological rhythm rather than fixed clock-time recommendations.

Chronotherapy in metabolic and endocrine disorders

Chronotherapy in Diabetes Management

Although still evolving, evidence suggests that aligning drug administration with circadian metabolic patterns may improve glycemic control. For example, evening dosing of certain long-acting agents may better target nocturnal hepatic glucose production and lipolysis, while morning dosing of rapid-acting insulin may optimize postprandial glucose handling. Furthermore, metformin activates the circadian-linked AMPK pathway; bedtime extended-release dosing may suppress overnight hepatic gluconeogenesis. It may help reduce the early-morning rise in blood glucose (the dawn phenomenon), although clinical evidence remains limited. Similarly, chronodynamic principles may apply to incretin-based therapies. Endogenous GLP-1 follows a circadian rhythm, with secretion often peaking in the afternoon. Aligning short-acting GLP-1 receptor agonists or dipeptidyl peptidase-4 (DPP-4) inhibitors with these peaks may theoretically enhance meal-related glucose control, although this strategy lacks clinical validation [19]. However, most of these timing strategies are based on mechanistic evidence and small studies, are not included in current diabetes guidelines, and require validation in adequately powered prospective trials before routine clinical use.

Metabolic Syndrome and Circadian Misalignment

Metabolic syndrome represents a state of circadian disruption at multiple levels, including hormonal imbalance, altered feeding patterns, and impaired sleep-wake cycles. Chronotherapeutic strategies aim to restore alignment through timed medication, structured feeding schedules, and behavioral circadian interventions. A prominent, evidence-based example of this is time-restricted feeding (TRF), which consolidates all caloric intake into an early 8-to-10-hour daytime window [19]. Early TRF acts as a potent synchronizer for peripheral clocks in the liver and adipose tissue, upregulating pathways like SIRT1 and AMPK to independently improve insulin sensitivity, lipid oxidation, and blood pressure, even in the absence of weight loss [1,19]. Such integrative approaches recognize that drugs alone cannot correct a system that is temporally disrupted.

Chronotherapy in Thyroid Hormone Replacement

Levothyroxine, the mainstay of hypothyroidism treatment, illustrates how administration timing can influence drug exposure. In a randomized, double-blind crossover trial, bedtime levothyroxine intake lowered serum thyrotropin by 1.25 mIU/L (95% CI: 0.60-1.89) and modestly increased free thyroxine and triiodothyronine compared with intake 30 minutes before breakfast, an effect attributed to enhanced overnight intestinal absorption during prolonged fasting [31]. However, subsequent randomized trials and a meta-analysis, including studies in older and drug-naïve patients, found no significant difference in thyroid-stimulating hormone control between bedtime and morning administration [32-34]. Bedtime dosing therefore represents a reasonable and often better-tolerated alternative for selected patients, although the evidence for a consistent biochemical advantage remains mixed rather than established; separation of levothyroxine from interacting drugs and food remains the more firmly supported timing principle.

Chronotherapy in respiratory diseases

Chronotherapy in Asthma

Several factors contribute to the nocturnal worsening of asthma, including reduced circulating cortisol and epinephrine levels during sleep, increased vagal tone leading to bronchoconstriction, circadian variations in inflammatory cytokines, altered airway epithelial function, and nocturnal recruitment of eosinophils and neutrophils regulated by clock genes such as BMAL1. Together, these circadian neuroendocrine and immune mechanisms increase airway hyperreactivity, causing nocturnal and early morning worsening of asthma symptoms [5,22]. To counteract this, chronotherapy delivers maximum drug effect when biological risk is highest. For example, evening ingestion of sustained-release theophylline utilizes its delayed absorption profile to achieve peak plasma concentrations (Cmax) overnight, maximizing chronoefficacy while minimizing daytime toxicity [21,22]. Similarly, evening administration of long-acting β2-agonists or prodrugs like bambuterol may align peak receptor engagement with the early-morning drop in forced expiratory volume (FEV1). Evening or late afternoon dosing of corticosteroids suppresses nocturnal cytokine production while minimizing hypothalamic-pituitary-adrenal (HPA) axis suppression [21]. Similarly, evening administration of leukotriene receptor antagonists (montelukast) helps counter the overnight surge in bronchoconstricting cysteinyl leukotrienes [22].

Chronotherapy in Allergic Rhinitis

Allergic rhinitis also follows a circadian pattern, with symptoms such as nasal congestion and runny nose often worsening in the early morning due to delayed immune responses and the effects of lying down during sleep. Studies show that taking H1-antihistamines in the evening improves morning symptom control while reducing daytime side effects such as sedation and dry mouth [22].

Chronotherapy in rheumatologic disorders

Rheumatoid arthritis (RA) exhibits a distinct circadian pattern in which severe morning stiffness and functional limitation are driven by a nocturnal surge in pro-inflammatory cytokines, particularly interleukin 6 (IL-6), which peaks during the early morning hours. Under normal conditions, cortisol exhibits a circadian rise in the early morning, counteracting inflammation [6]. In RA, this cortisol response is insufficient compared to cytokine production, leading to persistent inflammation during the night. Modified-release prednisone administered at night releases the drug during the early morning hours, coinciding with peak pro-inflammatory cytokine activity. Clinical studies have shown reduced morning stiffness, improved symptom control, and better alignment with inflammatory rhythms [6,15,35,36]. In the CAPRA-1 randomized trial, for example, night-time modified-release prednisone reduced the duration of morning stiffness by 22.7% from baseline, compared with 0.4% for immediate-release prednisone (a mean reduction of about 44 minutes; p = 0.045) [36].

Chronotherapy in psychiatric disorders

The disruption of neuropsychiatric circadian signaling, such as phase-delayed or blunted rhythms in melatonin and cortisol, is a core pathophysiological feature of mood disorders like depression and bipolar disorder. Consequently, chronotherapeutic approaches in psychiatry include the timed administration of antidepressants, light therapy to reset the circadian phase, and sleep phase manipulation. These strategies aim to restore circadian alignment, rather than simply suppress symptoms [1]. The blood-brain barrier (BBB) harbors an endogenous molecular clock that regulates the rhythmic activity of multidrug efflux transporters (such as P-glycoprotein). This creates daily variations in BBB permeability that may influence central nervous system (CNS) drug accumulation and the efficacy of antidepressants and mood stabilizers [1,2]. An example of rhythm-targeted chronopharmacology is agomelatine, which acts simultaneously as a melatonin (MT1/MT2) receptor agonist and a serotonin (5-HT2C) receptor antagonist. Due to its short half-life, evening administration aligns peak plasma concentrations with endogenous melatonin secretion, supporting resynchronization of the sleep-wake cycle and its antidepressant action; although the specific benefit of dosing time remains to be established [1].

Chronotherapy in cancer immunotherapy

Circadian rhythms heavily regulate immune-cell trafficking, cytokine secretion, antigen presentation, and T-cell activation. For example, circulating levels of effector CD8+ T cells, the primary drivers of the anti-tumor response, and natural killer (NK) cells typically peak during the daytime, whereas naive and memory T cells peak at night. These rhythmic processes influence the tumor immune microenvironment and dictate the body's response to immunotherapy [12].

A growing number of retrospective studies suggest that the timing of immune checkpoint inhibitor (ICI) administration may influence clinical outcomes. Morning (early time-of-day) administration of programmed cell death protein 1 (PD-1)/programmed death-ligand 1 (PD-L1) and cytotoxic T-lymphocyte-associated protein 4 (CTLA-4) inhibitors has been associated with progression-free survival, with some studies reporting up to a four-fold increase, compared with afternoon administration [12,37,38]. These survival benefits have been observed across multiple malignancies, including melanoma, non-small-cell lung cancer, and renal cell carcinoma. Because ICIs have long half-lives (ranging from 14 to 27 days), researchers suggest that the timing of the initial infusions (during the first two to four months before the drug reaches steady-state plasma concentrations) is the most critical window for establishing long-term immune activation [37].

These findings highlight the considerable potential of incorporating circadian timing into precision oncology, but individual variability may limit universal dosing recommendations. However, evidence is largely retrospective, and immune checkpoint inhibitor timing is not yet incorporated into oncology guidelines, requiring prospective randomized validation before routine practice. Future studies using digital circadian biomarkers may enable personalized chrono-immunotherapy aligned with each patient’s biological rhythms [12,37].

Table 1 summarizes these chronotherapeutic timing recommendations across the organ systems and disorders discussed above, together with the corresponding level of supporting clinical evidence for each.

**Table 1:** Comprehensive chronotherapeutic timing. Evidence Level: Established - supported by randomized trials, regulatory approval, or standard clinical practice. Emerging - limited or observational clinical evidence, not yet incorporated into guidelines. Preliminary - based mainly on mechanistic rationale or small studies, with clinical benefit unproven. Mixed - high-quality randomized trials conflicting or neutral; pharmacokinetic or physiological observation rather than a direct clinical timing recommendation. ACE: angiotensin-converting enzyme; ARBs: angiotensin receptor blockers; GLP-1: glucagon-like peptide-1; DPP-4: dipeptidyl peptidase-4; SR: sustained release; LABAs: long-acting beta agonists; GFR: glomerular filtration rate; PD-1: programmed cell death protein 1; PD-L1: programmed death-ligand 1; CTLA-4: cytotoxic T-lymphocyte-associated protein 4; BP: blood pressure; HPA: hypothalamic-pituitary-adrenal; RCT: randomized controlled trial.

System	Drug class/Biological process	Recommended timing	Chronotherapeutic rationale	Evidence level
Cardiovascular	ACE inhibitors, ARBs	Individualized (morning or bedtime)	May improve nocturnal BP control and restore dipping patterns; routine bedtime dosing remains controversial [26,27].	Mixed - RCTs neutral (TIME, BedMed)
	Aspirin, short-acting statins	Evening/bedtime	Targets early-morning platelet activation and nocturnal cholesterol synthesis [4,18].	Established (statins); Preliminary (aspirin)
Metabolic and endocrine	Rapid-acting Insulin	Morning/Pre-meal	Optimizes postprandial glucose control.	Established (standard pre-meal practice)
	Short-acting GLP-1 agonists, DPP-4 inhibitors	Afternoon	Aligns with physiological peaks in incretin activity and insulin secretion.	Preliminary (mechanistic)
	Extended-release metformin	Evening/bedtime	Reduces overnight hepatic gluconeogenesis and the dawn phenomenon [19].	Preliminary (limited clinical data)
	Levothyroxine	Bedtime or morning (individualized)	Enhanced overnight intestinal absorption during fasting [31].	Mixed (early RCT positive; later trials and meta-analysis neutral)
Immune/inflammatory	Corticosteroids	Late afternoon/evening	Suppresses nocturnal cytokine surges while minimizing HPA-axis disruption [21].	Emerging
Respiratory	Theophylline (SR), LABAs, montelukast, H1-antihistamines	Evening/bedtime	Counteracts nocturnal airway narrowing, leukotriene release, and early-morning symptoms [21,22].	Established
Rheumatologic	Modified-release prednisone	Bedtime	Timed release attenuates the early-morning IL-6 surge and reduces morning stiffness [6,15].	Established (RCT-supported, approved)
Neuropsychiatric	Agomelatine	Evening/bedtime	Synchronizes with endogenous melatonin secretion and circadian sleep-wake rhythms [1].	Established (per product labeling)
Gastrointestinal	Lipophilic drugs (absorption)	Morning	Enhanced absorption due to increased gastrointestinal blood flow and gastric emptying in the morning [10].	Preliminary (mechanistic)
Hepatic	CYP450-mediated phase I reactions	Daytime	Oxidative metabolic activity peaks during the active phase.	-
	Phase II conjugation reactions	Night-time	Conjugative metabolism is relatively enhanced during the rest phase [10].	-
Renal	GFR-dependent drug clearance	Daytime	Higher glomerular filtration rate and renal blood flow during the day, which increases clearance [3,8].	-
Biliary	Biliary excretion	Morning/early rest phase	Circadian variation in bile flow and transporter activity influences drug elimination [2,11].	-
Oncology	Immune checkpoint inhibitors (PD-1, PD-L1, CTLA-4 inhibitors)	Morning	Circadian variation in immune-cell activity and T-cell responsiveness may enhance antitumor efficacy; morning administration has been associated with improved survival outcomes in melanoma and lung cancer [12,37].	Emerging (retrospective; not yet in guidelines)

Advanced integration, drug delivery, and future directions

Chronopharmacokinetic-Pharmacodynamic (PK-PD) Modeling

Modern chronotherapy requires quantitative prediction of how circadian rhythms influence drug response. Chronopharmacokinetic-pharmacodynamic (PK-PD) modeling addresses this by incorporating circadian fluctuations in ADME processes and target sensitivity, enabling more accurate prediction of time-dependent therapeutic outcomes than traditional static models. These models embed circadian functions, typically sinusoidal terms such as cosine or Fourier series, into PK-PD equations to simulate rhythmicity and predict optimal dosing. Extensions of indirect-response (IDR) and target-mediated drug disposition (TMDD) models that incorporate rhythmic receptor turnover improve prediction of time-dependent effects and may support more individualized strategies [3,39].

Chronotherapeutic drug delivery systems

Chronotherapy requires not only identifying the optimal timing for drug action but also ensuring drug delivery coincides precisely with the desired biological time, particularly when symptom peaks occur during sleep or outside routine dosing schedules [2]. This challenge has led to the development of chronotherapeutic drug delivery systems, specifically designed to provide controlled and time-directed drug release [4].

Delayed-Release Drug Delivery Systems

These formulations introduce a lag period between drug administration and release. For example, a drug taken at bedtime may release four to six hours later [4,15]. This ensures peak plasma concentration coincides with early morning symptom peaks [6]. A classic example is modified-release prednisone in rheumatoid arthritis, where nocturnal administration allows glucocorticoid release during the early morning cytokine surge [6,15,35]. Another example is the Chronotherapeutic Oral Drug Absorption System (CODAS®), utilized in formulations like Verelan® PM (verapamil). When taken at bedtime, this pellet-filled capsule provides a four-to-five-hour delay in drug release to effectively suppress the sharp early morning rise in blood pressure and heart rate without being affected by food or posture [4,40]. Furthermore, drugs like Circadin® utilize delayed-release technology to mimic physiological nocturnal melatonin profiles, gradually releasing the hormone over five to seven hours following evening intake to treat sleep disorders [39].

Pulsatile Drug Delivery Systems

Pulsatile systems release drug in rapid bursts rather than continuously, suiting diseases with predictable symptom peaks [4,39]. Formulations engineered to counter early-morning asthma and allergic rhinitis include pulsatile-release montelukast capsules, multilayer theophylline-diltiazem tablets delivering two timed pulses, and "tablet-within-a-tablet" albuterol products (e.g., Proventil Repetabs) [22,39]. These rely on technologies such as the Pulsincap system (a swellable hydrogel plug ejected after a set lag) and osmotic programmable oral release technology (PORT) systems, while externally regulated platforms using ultrasound, magnetic, or electrical triggers enable on-demand release from implants or nanocarriers [40,41].

Controlled and Programmable Systems

Advances in drug delivery now allow programmable release profiles, multi-phase drug delivery, and chronoresponsive systems triggered by physiological signals. These technologies are gradually transforming chronotherapy from a simple dosing adjustment strategy into an engineered and individualized therapeutic intervention. For example, in the field of clinical oncology, programmable-in-time multichannel infusion pumps (such as the IntelliJect and Melodie devices) have been engineered to deliver circadian-chronomodulated chemotherapy. These pumps automatically vary the infusion flow rate, ranging from constant to sinusoidal over a 24-hour period to maximize the therapeutic index of multi-drug regimens (like 5-fluorouracil, leucovorin, and oxaliplatin) without requiring the patient to be hospitalized [39]. Additionally, cutting-edge bio-responsive systems are emerging, such as smart hydrogels and microneedle arrays that dynamically release insulin strictly in response to elevated physiological glucose levels, providing a highly personalized, stimuli-responsive chronotherapy [41].

Molecular targets in circadian pharmacology

Circadian rhythms not only influence drug handling but also molecular targets upon which drugs act. Increasing evidence suggests that many pharmacological targets themselves undergo rhythmic oscillation across the day [1,42].

Clock Genes as Therapeutic Targets

Beyond the physiological roles of the core clock genes described earlier, these genes are themselves emerging drug targets, offering novel therapeutic opportunities in cancer, metabolic, and inflammatory diseases. For example, synthetic small-molecule modulators such as KL001 (a CRY stabilizer) or SR9009 and SR9011 (reverse erythroblastosis virus (REV-ERB) agonists) have shown efficacy in preclinical models of diabetes, obesity, and glioblastoma by directly altering the circadian period and amplitude [12]. This represents a paradigm shift from adapting drugs to the clock (clocking the drugs) toward pharmacologically modulating the clock itself (drugging the clock) [42].

Barriers and practical challenges in chronotherapy

Despite growing understanding and accumulating clinical evidence, chronotherapy remains underutilized in routine clinical practice. The reasons are multifactorial.

Clinical Inertia and Conventional Prescribing Practices

Medical practice often relies on standardized dosing schedules, which are easier to implement but ignore biological variability [17]. In many medical settings, particularly hospitals, the timing of medication administration is dictated entirely by institutional routines rather than the patient's biological requirements [7,9]. As a result, therapeutic timing is commonly treated as a secondary consideration rather than an integral component of pharmacological optimization [17].

Lack of Guidelines

Most clinical trials overlook administration time, limiting evidence-based chronotherapeutic recommendations. Despite supporting evidence, therapeutic timing remains underrepresented in treatment protocols and prescribing information, highlighting the need for standardized chronotherapeutic guidelines and integration of chronobiology into medical education and clinical practice [17]. Notably, current chronotherapeutic recommendations focus largely on optimizing pharmacological interventions after disease onset. Because circadian disruption is itself a recognized risk factor for cardiometabolic and other chronic diseases, future chronobiology-guided guidelines could extend beyond medication timing to incorporate evidence-based lifestyle interventions, such as regular meal timing, sleep regularity, and appropriately timed physical activity, as an upstream strategy for disease prevention, complementing therapeutic chronotherapy [43,44].

Interindividual Variability

Circadian rhythms differ between individuals due to chronotype (morning vs. evening preference), lifestyle factors, and comorbid conditions [12]. Because chronotypes are heavily influenced by age, sex, genetics, environmental light levels, and seasonal changes, universally prescribing a medication at "8:00 a.m." on the wall clock does not correspond to the same biological morning for everyone [4,45]. Furthermore, individuals engaged in shift work or experiencing "social jetlag" suffer from a severe misalignment between their endogenous clocks and their lifestyle, meaning their optimal treatment windows will dynamically shift and differ drastically from those with standard sleep-wake routines [22,46].

Logistical and Practical Constraints

Practical implementation also presents important challenges. Timed dosing may be inconvenient for patients, particularly when optimal timing occurs during sleep or early morning hours. Additionally, adherence to strict "time-of-day" schedules constitutes a formidable challenge for patients managing polypharmacy, where multiple medications may each require a distinct, rigid dosing time [22]. For example, while studies strongly support the timed administration of chemotherapy to reduce toxicity, it is rarely implemented as standard practice because scheduling infusions at highly specific hours places a severe strain on hospital infrastructure and outpatient logistics [45]. In addition, certain chronotherapeutic strategies require specialized drug delivery systems capable of delayed or pulsatile release, which may not always be widely available or economically accessible [4]. Therefore, overcoming these practical barriers will rely heavily on the development and widespread adoption of programmable infusion pumps, advanced nanomaterials, and autonomous drug-delivery systems that do not require active patient compliance [22,45]. Thus, successful integration of chronotherapy into clinical practice requires not only scientific validation of PK-PD rhythms but also patient-centered and practically feasible therapeutic solutions [22].

Practical recommendations for clinical implementation

Current evidence supports only a limited number of chronotherapeutic strategies for routine use, including evening/bedtime dosing of short-acting statins, nighttime modified-release prednisone in rheumatoid arthritis, and appropriately timed controller therapy for nocturnal asthma and allergic rhinitis. Agomelatine is also administered in the evening according to its approved labeling. Antihypertensive timing should be individualized based on ambulatory blood pressure, tolerability, and adherence rather than routinely shifted to bedtime. Other strategies, including timing of GLP-1 receptor agonists, DPP-4 inhibitors, metformin, most metabolic and psychiatric therapies, and immune checkpoint inhibitors, remain investigational and require further randomized evidence before routine prescribing changes can be recommended.

The future of precision chronomedicine: systems chronotherapeutics

The future of chronotherapy lies in the development of personalized, data-driven therapeutic strategies capable of integrating circadian biology into individualized patient care. Advances in digital wearable health technologies, computational medicine, circadian biomarker analysis, and systems pharmacology are rapidly transforming chronotherapy from a conceptual framework into a clinically actionable precision-medicine approach known as systems chronotherapeutics [12,39]. Wearable devices can continuously monitor parameters such as sleep-wake cycles, body temperature, hormonal fluctuations, heart rate variability, and activity patterns, providing real-time insight into an individual’s biological clock [39,47]. This approach integrates molecular circadian biology, organ-level physiology, and drug kinetics and dynamics. This framework recognizes that drug response is not determined by a single variable, but by a complex network of oscillating systems interacting continuously over time. Such models are particularly relevant for complex diseases like cancer, cardiovascular disorders, and metabolic syndrome, where multiple interconnected pathways exhibit circadian regulation [12,39].

To account for interindividual differences in chronotypes and drug metabolism, future chronotherapy must move beyond fixed clock-time recommendations. Accurately assessing an individual’s internal circadian time is therefore essential. Currently, this relies on established clinical methodologies such as measuring the dim light melatonin onset (DLMO), the gold standard for determining the phase of the central pacemaker [4,48], or analyzing clock-gene expression from hair-follicle biopsies to reliably evaluate peripheral molecular rhythms [21]. Advances in wearable technologies further complement these methods by enabling continuous, non-invasive monitoring of physiological variables such as wrist temperature, heart rate, rest-activity cycles, and ambient light exposure. Emerging microfluidic wearables are also being developed to measure biochemical markers, including cortisol, interleukin-1β, and C-reactive protein, directly from sweat in real time [47]. By integrating these circadian biomarkers with longitudinal wearable-derived data, clinicians could, in principle, develop patient-specific “biorhythmic digital twins” [12]. These digital representations would combine artificial intelligence (AI) with multiscale physiologically based pharmacokinetic-pharmacodynamic (PBPK-PD) models to characterize individual circadian physiology. Wearable-derived data can reveal subtle circadian desynchronies [47]. Incorporating these real-time circadian parameters into AI-driven models could enable simulation of drug absorption, metabolism, and clearance across the 24-hour cycle. Such systems could facilitate dynamic dosing schedules that maximize chronoefficacy, minimize chronotoxicity, and support personalized chronodiagnosis [12,20]. At present, however, such AI-integrated “digital twin” systems remain largely conceptual; they have not yet been validated in clinical settings, and the supporting literature describes proposed frameworks rather than demonstrated clinical benefit.

More broadly, these developments point toward comprehensive circadian health management, which can be conceptualized as three complementary levels of chronobiological intervention: (i) preservation of circadian homeostasis through regular lifestyle practices, including consistent sleep-wake, meal, and activity schedules; [43] (ii) restoration of disrupted rhythms through behavioral chronobiological interventions such as time-restricted feeding and light therapy; [44] and (iii) precision chronotherapy through optimized medication timing [45].

Research gaps and future directions

Several areas require further exploration, such as large-scale clinical trials incorporating time-of-day variables, standardization of chronotherapy protocols, identification of reliable non-invasive circadian biomarkers, integration of chronotherapy into clinical guidelines, and development of accessible chronotherapeutic formulations such as programmable-in-time infusion pumps and rhythm-sensing drug-releasing nanoparticles [39]. Bridging these gaps will be essential for translating chronobiological insights into routine clinical practice and establishing time-optimized dosing as a fundamental pillar of personalized medicine [12]. Also, recent findings from the TIME and BedMed trials underscore the importance of validating chronotherapeutic strategies through large, well-designed randomized clinical trials before widespread clinical implementation [26,27]. A further priority is the development and validation of comprehensive, chronobiology-guided care models that integrate preventive and therapeutic circadian interventions. Future studies should examine whether combining precision pharmacological chronotherapy with evidence-based lifestyle interventions, such as personalized meal timing, sleep optimization, timed physical activity, and environmental light synchronization, improves metabolic and clinical outcomes beyond medication timing alone, thereby providing an evidence base for comprehensive chronobiology-guided clinical recommendations.

## Conclusions

Circadian biology has emerged as a critical determinant of both disease expression and therapeutic response, providing a scientific foundation for the optimization of pharmacotherapy through chronopharmacology and chronotherapy. Evidence across cardiovascular, respiratory, rheumatologic, metabolic, and psychiatric disorders demonstrates that aligning drug administration with endogenous biological rhythms can enhance efficacy, reduce adverse effects, and improve treatment outcomes. However, the strength of this evidence varies considerably: it is robust for interventions such as evening dosing of short-acting statins, modified-release prednisone in rheumatoid arthritis, and selected chronomodulated chemotherapy regimens, whereas it remains mixed or evolving for others, including bedtime antihypertensive dosing and many metabolic and psychiatric applications. Advances in circadian biomarker assessment, wearable technologies, computational modeling, and precision medicine are further accelerating the transition from population-based dosing strategies to individualized, time-directed therapy. Although challenges remain, including interindividual variability in circadian rhythms and the need for standardized clinical guidelines, the integration of biological time into therapeutic decision-making represents a promising evolution in modern medicine. Further high-quality randomized controlled trials that incorporate time of administration as a study variable are needed to define optimal timing strategies before universal chronotherapeutic recommendations can be made. In addition to optimizing medication timing, evidence-based lifestyle interventions that help preserve and restore circadian homeostasis may complement precision chronotherapy and represent an important direction for future research. Such an integrated perspective, uniting pharmacological and non-pharmacological circadian strategies, would further strengthen the relevance of chronomedicine to precision healthcare and to future guideline development. Ultimately, as high-quality randomized evidence accumulates, incorporating the dimension of time into pharmacotherapy has the potential to refine treatment paradigms and advance the goal of truly personalized medicine, though universal recommendations across all disease areas are not yet warranted.
